# Multi-level aspect based sentiment classification of Twitter data: using hybrid approach in deep learning

**DOI:** 10.7717/peerj-cs.433

**Published:** 2021-04-13

**Authors:** Sadaf Hussain Janjua, Ghazanfar Farooq Siddiqui, Muddassar Azam Sindhu, Umer Rashid

**Affiliations:** Department of Computer Sciences, Quaid-i-Azam University, Islamabad, Pakistan

**Keywords:** Aspect-based sentiment classification, Feature extraction, Feature selection, Hybrid approach, Information gain, Multi-layer perception, Principal component analysis

## Abstract

Social media is a vital source to produce textual data, further utilized in various research fields. It has been considered an essential foundation for organizations to get valuable data to assess the users’ thoughts and opinions on a specific topic. Text classification is a procedure to assign tags to predefined classes automatically based on their contents. The aspect-based sentiment analysis to classify the text is challenging. Every work related to sentiment analysis approached this issue as the current research usually discusses the document-level and overall sentence-level analysis rather than the particularities of the sentiments. This research aims to use Twitter data to perform a finer-grained sentiment analysis at aspect-level by considering explicit and implicit aspects. This study proposes a new Multi-level Hybrid Aspect-Based Sentiment Classification (MuLeHyABSC) approach by embedding a feature ranking process with an amendment of feature selection method for Twitter and sentiment classification comprising of Artificial Neural Network; Multi-Layer Perceptron (MLP) is used to attain improved results. In this study, different machine learning classification methods were also implemented, including Random Forest (RF), Support Vector Classifier (SVC), and seven more classifiers to compare with the proposed classification method. The implementation of the proposed hybrid method has shown better performance and the efficiency of the proposed system was validated on multiple Twitter datasets to manifest different domains. We achieved better results for all Twitter datasets used for the validation purpose of the proposed method with an accuracy of 78.99%, 84.09%, 80.38%, 82.37%, and 84.72%, respectively, compared to the baseline approaches. The proposed approach revealed that the new hybrid aspect-based text classification functionality is enhanced, and it outperformed the existing baseline methods for sentiment classification.

## Introduction

Sentiment analysis (SA) is presently a challenging topic mainly dealt with Natural language processing (NLP). It explores people’s sentiments, beliefs, appraisals, attitudes, feelings, and assessments regarding the objects for instances, products, systems, services, affairs, proceedings, subjects, entities, and their attributes (Liu & Zhang, 2012). The document-level SA is the most common form of SA, and it uses different ways to find document-level and sentence-level polarities. The document-level approach considers the entire document to find the sentiments, and there may be several opinions and reviews about a single entity. On the contrary, sentence-level SA encompasses the sentiment analysis of a single sentence in a document. Different entities can be measured in sentence-level SA while considering the whole document. Before analyzing the polarity in sentence-level SA, the essential thing to consider is that the sentence belongs to either a subjective category or an objective category. Only the subjective sentences can be considered to find the polarity (Ding, Liu & Yu, 2008).

The techniques used to find the overall polarity of any document or sentence will not give accurate results. The same is the case with document-level SA. It does not consider each aspect in the document, and the algorithm generates results based on overall polarity like positive or negative. The aspect-level SA considers the aspects in the sentence, and a sentence can have multiple aspects like price, quality, color, weight, etc. Not all the aspects need to have positive or negative opinions. Mostly in a sentence, some aspects can have positive, and some aspects can have negative reviews. It is essential to consider that a document or a sentence may consist of a positive opinion and it does not lead to the conclusion that people have only positive opinions or reviews about each aspect of that particular entity. The same is the case with negative opinions (Liu et al., 2005). So, such type of data is not correctly classified using document and sentence-level SA models. For more accurate results, there is a need for some fine-grained model, which can extract detailed opinions of each aspect of an entity in a sentence precisely.

Aspect-Based Sentiment Analysis (ABSA) is used for fine-grained sentiment analysis that considers the target entity and is determined as a research problem. In a document, ABSA is used to express an entity’s aspects by identifying each aspect; the polarity of sentiment is measured using a specific approach (Feldman, 2013). The ABSAs’ primary purpose is to extract the relevant opinionated aspects and then classify them into different categories like “positive”, “negative”, and “neutral”. Moreover, document-level and sentence-level sentiment analyses do not precisely regulate the target entity’s aspects’ polarities. They cannot figure out precisely people’s choices as likes or dislikes.

Aspect-based feature extraction and sentiment classification are two main tasks of ABSA. The main aspects of an entity are identified in the task of feature extraction. In the various ABSA studies, feature extraction is determined by nouns, noun phrases, or noun groups (Liu et al., 2005). Park & Kim (2016) proposed a hybrid mechanism with the fusion of two methods, machine learning algorithms, and lexicon-based approaches. They used lexicons like Senti-WordNet for sentiment word detection and POS tagging related to feature selection (Park & Kim, 2016).

Wu et al. (2018) proposed a hybrid unsupervised approach for Opinion Term Extraction (OTE) and Aspect Term Extraction (ATE) by combining rule-based, and machine learning approaches. Yan et al. (2015) used a Node Rank method to extract explicit and implicit aspects. Su et al. (2006) used point-wise mutual information (PMI) to extract the implicit aspects. Only explicit features were extracted, and there was no context in implicit sentences. Zainuddin, Selamat & Ibrahim (2018) suggested a hybrid approach for classification by using dependency parser for implicit aspects and POS tagging for explicit aspects. De Clercq et al. (2015) presented a model LT3 in which they first derive the aspect term and then classified them into polarities and different categories. They used different lexicon and semantic features to derive aspects.

The discussed methods can work well on the aspects that are intensely related to specific categories of words like (nouns); however, these methods often fail for the low-frequency words used as aspects. There must be a unique possible combination of these approaches that can be used to classify context-dependent opinions precisely. Despite that, all the previously proposed models still need improvement to classify the sentiments at the aspect-level.

The goal of this research work is to classify aspect-based sentiments using a hybrid approach. The proposed approach employs neural networks/deep learning instead of traditional machine learning classifiers to gain more accurate and efficient results. In the proposed research work, we suggest a new method based on multi-level hybrid aspect-based sentiment classification using Twitter attributes as features to maximize ABSA’s functionality. Our proposed system was validated on Twitter datasets including STC dataset (Saif et al., 2013; Zainuddin, Selamat & Ibrahim, 2018), TAS dataset (Wan & Gao, 2015), FGD dataset (Ankit & Saleena, 2018), ATC dataset (Kaur, 2017) and STS dataset (Go, Bhayani & Huang, 2009; Zainuddin, Selamat & Ibrahim, 2018). Four activation functions were implemented on all five datasets to estimate the functionality of the proposed system. The main contributions of the proposed approach are the following:

- We enhanced the accuracy in determining the explicit and implicit aspects at multi-level terms considering the aspects based on a single word and multi-word by using an approach that uses Association Rule Mining (ARM) with the fusion of POS patterns plus Stanford Dependency Tree (SDT).
- We improved the performance of the hybrid aspect-based sentiment classification method, which comprises a rule-based technique for detecting sentiment words, IG for feature ranking, and PCA to select ranked features by incorporating several activation functions to work with the MLP classification method and examined its performance.
- We employed other classification algorithms in testing which include, K Nearest Neighbors classifier (KNN), Logistic Regression (LR), Support Vector classifier (SVC), Decision Tree classifier (DT), Gaussian NB (NB), Random Forest classifier (RF), Ada Boost classifier (AB), Gradient Boosting classifier (GB), Extra Tree classifier (ET) and our proposed model by incorporating deep learning method: Multi-Layer Perceptron (MLP).
- We analyzed the influence of a hybrid approach on the neural network by applying various algorithms based on machine learning, which was adopted to test the accuracy.
- We focused on various dataset sizes and domains to evaluate the proposed system and it showed better results as compared to the existing methods independent of domain and size in all the datasets (used in this research work).

The rest of the paper is organized as follows: “Literature Review on Sentiment Analysis” provides a review of the related literature on ABSA. “Proposed Multi-Level Hybrid Text Classification Approach” discusses the proposed model based on a hybrid classification of sentiments and their structure. “System Evaluation and Results Discussion” describes the evaluation framework, the datasets used, the experiments done, and the results obtained from the experiments. “Conclusions and Future Directions” discusses the conclusion and highlights of future research directions.

## Literature review on sentiment analysis

The researchers presented several methods and approaches for text classification in aspect-based sentiment analysis (ABSA) (Liu, 2012). Specifically, three types of traditional approaches are used for the classification of “opinions” in SA, which are lexicon-based approaches, machine learning approaches, and the combination of preceding two approaches that are termed as hybrid strategies. The following are the major approaches for sentiment analysis.

### Lexicon based approaches

Lexicon-based approaches use dictionaries like WordNet and Senti-WordNet (Miller, 1995), there is no need for a training dataset, and the terms are used for scoring the sentiment from range −1 to 1. The term relates to a single word, phrase, or expression (Chiavetta, Bosco & Pilato, 2016). Mowlaei, Abadeh & Keshavarz (2020) addressed a problem of reduced performance of aspect-based methods due to the failure to adapt general lexicons of datasets based on aspects. To address this issue, an extension is proposed of two lexicon-generation procedures. In the lexicon-based approach, the performance is noticeably declined with the increase in the dictionary’s size and in classifying the context-dependent opinions precisely.

### Machine learning approaches

Unlabeled data is used in the unsupervised machine learning method. Rothfels & Tibshirani (2010) used this approach to measure movie reviews’ sentiments, but results were not satisfactory as they increased the word list. In the supervised machine learning method, labeled data is used for the training process and many algorithms like Maximum Entropy (ME), Naive Bayes (NB), and Support Vector Machine (SVM) are used in this approach. These are the most common and straightforward models used for text classification purposes (Tang, Kay & He, 2016). Chi-Square and Information Gain are feature selection approaches that are used to achieve greater accuracy in SA. Kastrati, Imran & Kurti (2020) proposed a feedback framework utilizing the strategy of weak supervision to estimate the aspect categories. Further, the attitude was examined with the help of aspects using comments. However, SVM is not as good as maximum entropy (ME) and NB because of its time complexity.

### Artificial neural network

Various types of Artificial Neural Networks (ANNs) have been used for SA by different researchers. Kalchbrenner, Grefenstette & Blunsom (2014) presented a Dynamic Convolutional Neural Network (DCNN) in which a Dynamic K max-pooling operator is used and the author performed some experiments with some variations of CNN. In another work, Socher et al. (2013) used this model to get competitive results as compared to DCNN. Do et al. (2019) have done an inclusive overview of main deep learning methods for aspect-level sentiment analysis, including LSTM, CNN, and many others. The authors suggested that better results can be obtained by extracting and using the classification of the aspect, sentiment, and category. Liu & Shen (2020) projected a distinctive neural network model named Gated Alternate Neural Network (GANN) to address some limitations in the previously proposed models regarding noise in capturing the significant sentiment expressions and achieved better results.

#### Multi-layer perceptron

Akhtar et al. (2017) proposed a technique to combine deep learning and feature-based methods using MLP for financial sentiment analysis. They developed multiple models of deep learning based on LSTM, GRU, and CNN. For training purposes, lexicon features and word embeddings are used. The proposed system showed good results on news and microblogs datasets (Akhtar et al., 2017). In another work, Pan, Hou & Liu (2009) used a hybrid approach using MLP for localizing the text and demonstrated good results. Ay Karakuş et al. (2018) evaluated several deep learning model performances in training and testing phases by constructing variants of models through changing layer sizes and word embedding process. They used LSTM, CNN, BILSTM, CNNLSTM and achieved improved results. Besides this, they also used MLP for classification purposes and gained testing accuracy of 78% on movie review dataset (Ay Karakuş et al., 2018). In another paper, the IDMB review dataset is used for sentiment classification, and MLP is used for the training process and it obtained good results (Hong & Fang, 2015). Most work related to sentiment classification in deep learning has been done on review datasets by achieving better results. There should be a hybrid approach to perform aspect-based sentiment classification of Twitter datasets to achieve maximum results in the testing phase using such a deep learning approach that needs to be improved in terms of accuracy.

### Sentiment classification using hybrid approach

Bansal & Srivastava (2019) proposed a hybrid method based on attributes that analyze customers’ intelligence by distinguishing aspects in the text aspects described using POS tags. Ma et al. (2018) proposed targeted-aspect-based classification with common-sense knowledge using Long and Short Term Memory (LSTM). They extend Sentic LSTM by adding a hybrid of LSTM and recurrent addictive network. In another research work, Wang, Xu & Wan (2013) extracted implicit features using hybrid ARM and they used five techniques to extract the features. Implicit words have no context attention, so only explicit aspects were extracted. Zainuddin, Selamat & Ibrahim (2018) used PCA+SVM with the combination of POS tags as feature extractors and obtained accuracy for three datasets as 71.62%, 76.55% for the STS dataset, and 74.24% for the STC dataset, respectively. This research’s main goal was to propose a new method based on multi-level hybrid aspect-based sentiment classification in neural networks using Twitter attributes as features to maximize the functionality of ABSA.

## Proposed multi-level hybrid text classification approach

**Figure 1:**
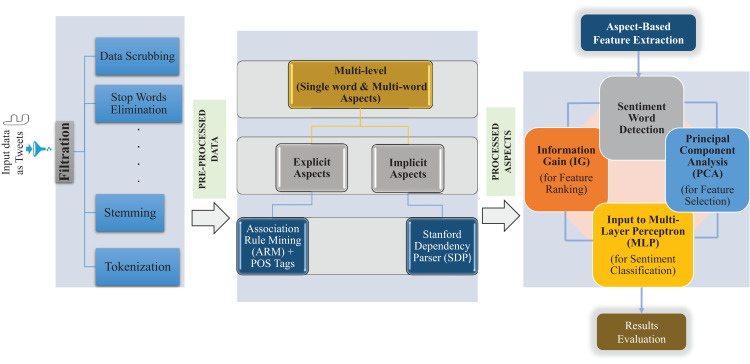
Proposed multi-level hybrid aspect-based sentiment classification (MuLeHyABSC) model.

We propose a novel technique for aspect-based sentiment classification. Our approach collects a Twitter dataset and performs text pre-processing. Further, our approach extracts feature to find the aspects based on multi-level single and multi-words by comprising of ARM method to detect implicit and explicit aspects with the fusion of the Stanford Dependency Tree (SDT) approach and POS patterns. For the sentiment word detection, aspect-based sentiment classification based on the hybrid method contains a rule-based approach, Information Gain (IG) for feature ranking process, and Principal Component Analysis (PCA) was incorporated for the selection of ranked features, and finally MLP for classification of sentiments. A detailed framework of a multi-level single word and multi-word aspect-based classification of Twitter data using a hybrid approach is shown in Fig. 1.

### Data gathering

We used a total of five datasets in this study, which include: Sanders Twitter Corpus (STC), Twitter Airline Sentiment (TAS), First GOP Debate (FGD), Apple Twitter Corpus (ATC), and Stanford Twitter Sentiment (STS). The STC dataset is shown in Table S1 and has been used by Zainuddin, Selamat & Ibrahim (2018). It comprises four categories “Apple”, “Google”, “Microsoft” and “Twitter” and we considered 1,091 tweets for the experiments. It can be downloaded from this link (http://www.sananalytics.com/lab/twittersentiment/). The second dataset TAS is shown in Table S2 and consists of five categories. It has 1,832 positive tweets and 5,741 negative tweets; the rest of the tweets were simply ignored. The FGD dataset is shown in Table S3, and it consists of eight categories. The ATC dataset is depicted in Table S4 and consists of only one category, “Apple”. The STS dataset shown in Table S5 used by Zainuddin, Selamat & Ibrahim (2018) was collected using the KNIME tool (Minanovic, Gabelica & Krstić, 2014). It has seven categories in which 357 tweets were considered. These datasets are very popular and used by many researchers for the evolution of Twitter sentiment classification. In the Twitter sentiment analysis, the use of all these datasets in the proposed approach has shown that the proposed method is independent of the domain in the detection of multi-level explicit and implicit aspects. We manually grouped and classified tweets into 3 categories as: “positive”, “negative”, “neutral and irrelevant” tweets in all the current study datasets. However, in the aspect-based text classification task, only positive tweets and negative tweets were considered for experimental purposes. A summary of the datasets regarding tweets is depicted in Table 1 and all the detailed Tables (related to this research work) can be found here (https://doi.org/10.5281/zenodo.4444759).

**Table 1:** Detailed number of Tweets in STC, TAS, FGD, ATC & STS datasets.

Sr No.	Dataset	Positive Tweets	Negative Tweets	Considered Tweets
1	STC	519	572	1,091
2	TAS	1,832	5,741	7,573
3	FGD	1,171	3,186	4,357
4	ATC	423	1,219	1,642
5	STS	180	177	357
Total		4,125	10,895	15,020

### Data scrubbing and transformation

Raw data consists of so many irrelevant attributes like URLs, links, usernames, retweets, emoticons, smilies, stop words, and reverting words used in the process of aspect-based text classification tasks and are considered useless. Consequently, it is needed to pre-process the tweets before analyzing them so that all the irrelevant attributes are removed from the datasets to avoid the contradiction of results. In this research, we have pre-processed all the datasets equally at multiple stages as described in the literature (HaCohen-Kerner, Miller & Yigal, 2020) and got improved results. Text pre-processing includes data cleansing by removing the unrelated data, including URLs, stop words, smilies, slang, redundant data, and all other irrelevant material. There are eight steps used to clean the data, including garbage removal (URLs, links, web addresses, etc), stop word elimination, slang rectification, case consistency, redundant data deletion, stemming, lemmatization, and tokenization.

### Aspect-based feature extraction

In this process, a group of features was selected using some ways to condense the dimension of feature space (Trier, Jain & Taxt, 1996). In the current research, aspect-based feature extraction was used to precisely extract the aspects based on a single word and multi-words. Specifically, two types of aspects known as explicit and implicit aspects were extracted using the feature extraction technique. Explicit aspects were extracted using ARM combining with POS tags. In contrast, the Stanford Dependency Parser (SDP) was only used to extract implicit aspects by determining the relationships between opinions and aspects.

#### Extraction of explicit multi-level (single and multi-word) aspects with association rule mining

In the proposed approach, ARM was used for finding explicit aspects and the data was analyzed for repetitive “if/then” patterns. There are two parameters to identify the most important relationships, which are Support and Confidence. Supporting criteria were used to identify how many times an item appeared in the database, while confidence was used to show how many times the “if/then” statement was true. A single word and multi-word aspects were extracted using ARM. The main issue with ARM is that it considers and generates all the association rules in rules set that have greater confidence and support than user-defined minimum confidence and support. To tackle this problem, in this research work, we considered an aspect to be important if it appeared in more than 1% of the sentences. ARM is based on the Apriori algorithm in which the frequency of aspects was found from a set of transactions that fulfilled the specification of the user for minimum support. Table S6 (http://doi.org/10.5281/zenodo.4444760) shows POS tags for single word aspects employed by literature (Socher et al., 2011).

At the experiment stage, various minimum support and minimum confidence values were executed. An appropriate minimum support value was considered as 0.1 and the value for minimum confidence was 0.5. In the primary experiment, only single word aspects from a noun and noun phrase using Association Rule Mining were detected. Furthermore, a heuristic combination of POS tags was applied to detect multi-word aspects from tweets. Table S7 (http://doi.org/10.5281/zenodo.4444760) shows POS tags (in heuristic combination) for the detection of multi-word aspects.

In the experiment of single word explicit aspects generation using singular and plural noun and proper noun patterns “NN NNS NNP NNPS”, some extracted phrases were “google”, “united”, “apple”, “lebron” and “nike” from multiple datasets. Using NNP RBR pattern, “united flight” was extracted, whilst “gop debate” with NN VBG pattern, was extracted. Besides this, using pattern NN JJ, the phrase “apple iphone” was extracted. In contrast, in the generations of multi-word explicit aspects, for instance using (DT-JJ) pattern e.g., aspects from TAS dataset: “jet blue flight”, using (NN-VB) pattern e.g., aspects from ATC dataset: “apple phone supremacy” was extracted. In addition, using (NN-JJ) pattern e.g., aspects from STC dataset: “twitter api”, “operating system”, “microsoft browser”, aspects from TAS dataset: “us airways”, “virgin america”, some aspects from FGD dataset: “ted cruz”, “black vote”, “jeb bush”, “donald trump”, “supreme court” and aspects from STS dataset: “time warner”, “san francisco”, “kindle 2” and “malcolm gladwell” were extracted. Table S8 (http://doi.org/10.5281/zenodo.4444760) shows some experimental results of single and multi-word detected explicit aspects from the STS dataset.

#### Extraction of implicit aspects using stanford dependency parser

Implicit aspects are not directly expressed in the sentence, and usually, they are not easy to extract when contrasted with explicit aspects. These aspects need to be found using relationships among opinions and aspects in a sentence. In this work, the Stanford Dependency Parser (SDP) was used to find a relationship to extract implicit aspects. Dependency parser and grammatical association were used to define implicit aspects. These aspects were determined using relations discovered by different types of dependencies. The sample of dependencies used for implicit aspects is depicted in Table S9 (http://doi.org/10.5281/zenodo.4444760).

In the process of extraction of implicit aspects, we took this tweet. Example: “The tooth pain got stale”, the patterns were found as: <The DT; tooth NN; pain NN; got VBD; stale JJ>—and the extracted aspects were nsubj(got, pain), dobj(got, stale) and compound(pain, tooth). The path “nsubj-advmod” was incorporated by a pair of verb (VB) and substantive, the paths “nsubj-dobj”, “amod”, “nsub-comp” and “nsubj-xcomp” were represented by a pair of adjective (JJ) and substantive (NN). For example, a sentence of tweet: “The tooth pain got stale”, an opinion pair was extracted <stale NN; pain JJ> by dependency path “amod”. Besides this, in the sentence: “had really bad tooth pain”, <had VBN; really RB; bad JJ; tooth NN; pain NN>, the extracted implicit aspect by dependency path “advmod” was, (bad, really) and the aspect got from example 1 was (pain, tooth), using compound dependency path that belongs to Misc (dentist) category of STS dataset. This process used transitive and direct dependencies in which we considered some important implicit aspects from each sentence to extract new possible implicit aspects from the previous sentence with the distance of 1.

In another example: “The treatment effects are still evident”. <The DT; treatment JJ; effects NNS; are VBP; still RB; evident JJ>—the extracted aspects by several dependency paths retrieved as: amod(effects, treatment), nsubj(evident, effects), advmod(evident, still), these aspects were extracted using “amod”, “nsubj” and “advmod”. In another example: we found a modifier relation (negation) that changes the sentiment of the tweet to the opposite as shown here, in this sentence: “This person is not helping”, POS patterns were detected as: <This DT; person NN; is VBZ; not RB; helping VBG>—and implicit aspects were extracted using “nsubj” as (helping, person) and “neg” dependency path will make this sentence negative immediately with the extraction of the aspects as: (helping, not). Tables S10–S14 (http://doi.org/10.5281/zenodo.4444760) shows some experimental results of detected implicit aspects for STC, TAS, FGD, ATC, and STS datasets, respectively.

### Hybrid text classification

This section describes the hybrid text classification method used in the proposed approach, uses a rule-based method to find sentiment words, detects feature opinionated words, and uses a classification algorithm for classifying the sentiments.

#### Sentiment word detection using rule based method

After the feature extraction process, the rule-based method was employed to detect the remaining aspects that were not identified by SDP. The essential purpose of using this technique was to find the aspect, sentiment word, and polarity values for each sentiment word. Algorithm 1 represents two rules used for this purpose.

**Algorithm 1:** Rule Based method used for detection of sentiment words.

**for** each sentence in the tweets **do**
Read the aspect in the sentence
**if** the aspect match **then**
Get sentiment word
**if** sentiment word distance less than or equal to 4 **then**
Add sentiment word into Results
**else**
Remove sentiment word
**end if**
**else**
Display no sentiment word
**end if**
**end for**
**for** each sentiment word in the Results **do**
Compute the sentiment word value
Display sentiment word value
**end for**

We considered an example to explain aspect extraction using the SDP method incorporated with grammatical relations. In the following tweet taken from STS dataset—Misc. “exam” category: “Worked harder, now only one exam left and I feel so happy, will have fun soon, anxiously waiting”. Initially, two aspects were extracted using the SDP method, first incorporating the relation “aux”, extracted aspect was “will fun”. Further, from another relation with SDP method “xcomp”, extracted aspect was: “feel happy”. In the first step, only the most important aspects would be considered and extracted. Three main points worked in this process: first, it depends on the sentiment word, and secondly, the position of the aspect that needed to be extracted from the tweet, and lastly, negative and positive values based on the polarities for all the labeled sentiment words were also considered.

#### Feature selection of sentiment words

For this proposed study, Information Gain (IG) was used for the feature ranking process, and Principal Component Analysis (PCA) was selected as a feature selection technique due to its effectiveness and better working. Although there are numerous feature selection methods such as Latent Semantic Analysis (LSA), Genetic Algorithms (GA), and many others. The findings in Vinodhini & Chandrasekaran (2014) show that PCA performs better for the feature selection procedure. The PCA is a multidimensional feature reduction technique that has the following steps: First, it converts the training dataset into the statistical dataset; Then it searches the covariance matrix; After this, it computes the Eigenvalues and Eileen vector for the covariance matrix; The next step is to sort the vector and values; In the end, the top vector is kept and trained, to test and analyze the reduced dataset.

#### Classification algorithm

**Figure 2:**
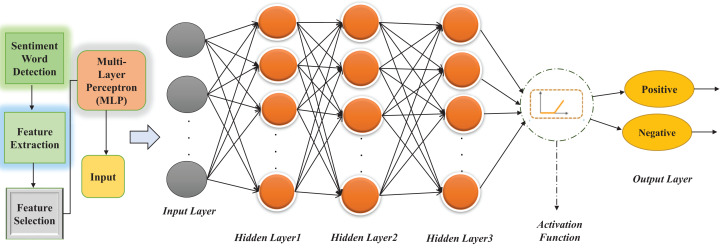
Proposed architecture of Multi-Layer Perceptron (MLP) for MuLeHyABSC.

After applying the Feature Selection (FS) technique, the next step is to classify the text using classification algorithms. For the current study, different machine learning algorithms were used to test the proposed system’s accuracy. However, Multi-Layer Perceptron (MLP), based on feed-forward ANN, outperformed and gave the highest accuracy when tested. It consists of multiple layers with 1 layer for input and 1 output layer with at least one hidden layer (there can be multiple hidden layers). Feed-forward means data flow in one form from the input layer to the output layer. In MLP, each layer of input is a predictor variable, and the neurons of one layer are linked to the neurons of the next layer, named the hidden layer(s). Each hidden layer’s neurons are connected to the next hidden layer except the last hidden layer, whose neurons are connected to the output layer. The output layer is created with one neuron when the prediction is binary and with N neurons when the prediction is non-binary (Singh & Husain, 2014). In this proposed MLP architecture, we have used a maximum of three hidden layers with the different numbers of neurons and 4 activation functions. The proposed architecture of MLP is shown in Fig. 2.

#### Activation functions

Different activation functions were used for the sentiment classification purpose, and these functions were used to convert the input to the hidden layers. These activation functions with multiple layers were implemented to test the accuracy for comparison purposes to observe which function gave optimal results. There is no rule for selecting the best function. It depends on the model, parameter, and features. In this case, there are minor differences in time and efficiency of different activation functions. These activation functions are explained as follows.

We reviewed different types of activation functions found in the literature Akhtar et al. (2017) and used them in this study to compare the results of our research with other similar studies. Identity (Eq. (1)) is a linear function and that’s why the output is not between any ranges. Logistic or Sigmoid (Eq. (2)) is like an S-shaped curve, and the range of this function is between 0 and 1. This activation function is not zero centered, and there is a problem with the vanishing gradient. Hyperbolic Tangent (Tanh) (Eq. (3)) activation function is better than the logistic function because it has a range between −1 and 1 as the output of this function is zero centered. The optimization of this function is easy. Therefore, it can choose over sigmoid or logistic function, but there is still a vanishing gradient problem. Rectified Linear Units (ReLU) (Eq. (4)) activation function has overcome the vanishing gradient problem. It is a simple and efficient activation function. Most deep learning models use this type of function due to its simplicity and efficiency.

#### Identity

(1) $$\begin{array}{r} f(x)=x \end{array}$$

#### Logistic or Sigmoid

(2) $$\begin{array}{r} F(x)=1/1+exp(-x) \end{array}$$

#### Hyperbolic Tangent function (Tanh)

(3) $$\begin{array}{r} f(x)=1-exp(-2x)/1+exp(-2x) \end{array}$$

#### Rectified Linear Units (ReLU)

(4) $$\begin{array}{r} R(x)=max(o,x) \end{array}$$

It means that R(x)=0 if R<0 and R(x)=x if R≥0

### Experimental setup

The proposed model, MuLeHyABSC+MLP was tested on five datasets namely STC, TAS, FGD, ATC, and STS shown in Table 1, which were considered as the benchmark datasets for multi-level single word and multi-word aspect-based text classification problem. The datasets contain tweets related to different topics (http://doi.org/10.5281/zenodo.4444760). The latest version of Python software was used for the experimental setup. It is a standard environment with state-of-the-art modules which was widely used for text classification purposes. Pandas, apriori, nltk, text blob, and sci-kit-learn are basic libraries used for testing the proposed model. At the experiment stage, various values based on minimum support and confidence were executed. Suitable values for minimum support and minimum confidence were considered to be 0.1 and 0.5 respectively.

## System evaluation and results discussion

Five datasets were considered for the experimental purpose as discussed in “Proposed Multi-Level Hybrid Text Classification Approach” including the STC dataset, Table S1, which consists of total of four categories, named as: “Apple”, “Google”, “Microsoft” and “Twitter”; TAS dataset, Table S2, with five categories named as: “Virgin America”, “United”, “South West Air”, “Jet Blue” and “US Airways”; FGD dataset, Table S3, which has eight categories named as: “Ben Carson”, “Chris Christie”, “Donald Trump”, “Jeb Bush”, “John Kasich”, “Marco Rubio”, “Mike Huckabee” and “Ted Cruz”; ATC dataset, Table S4, consists of one category named as: “Apple” and STS dataset, Table S5, with seven different main categories, namely: “Company”, “Person”, “Movie”, “Product”, “Location”, “Misc” and “Event”. In the first experiment, only single word explicit aspects were achieved from a noun and noun phrase using Association Rule Mining (ARM) as shown in Table S6. ARM and heuristic combination of POS tags were combined to use for multi-word aspects at multi-level as shown in Table S7. Table S8 represents some results of the experiments of aspects detection for the STS dataset. It shows that our proposed system successfully detected aspects from each category in different datasets, aspects such as: “cheney”, “north”, “yankees”, “yahoo”, “jquery”, “safeway” and “startrek” which were completely related to this research work. Furthermore, we achieved satisfying results in the detection of multi-word aspects using ARM with a heuristic combination of POS tags. Tables S10–S14 (http://doi.org/10.5281/zenodo.4444760) shows the summary of detected implicit aspects using grammatical relation of dependency parser shown in Table S9 for STC, TAS, FGD, ATC, and STS datasets, respectively.

### Evaluation criteria

The complete classification performance was measured using different evaluating parameters and this classification consists of binary values (positive and negative). Two ordinary evaluation measures precision (Eq. (5)) and recall (Eq. (6)) were used for the evaluation of the sentiments of the tweets based on positive and negative polarity including an accuracy measure of (Eq. (7)) and F-measure was used for the micro-averaging purpose (Eq. (8)). Four functional accuracy measures were taken into account based on the outcomes of the confusion matrix named true positive (TP), false positive (FP), true negative (TN), and false negative (FN). The evaluation parameters which were employed for measuring the performance of our proposed system are listed below:

#### Precision

(5) $$\begin{array}{r} TP/(TP+FP) \end{array}$$

#### Recall

(6) $$\begin{array}{r} TP/(TP+FN) \end{array}$$

#### Accuracy

(7) $$\begin{array}{r} \left(TP+TN)/(TP+FP+TN+FN\right) \end{array}$$

#### F-measure

(8) $$\begin{array}{r} 2*(Precision*Recall)/(Precision+Recall) \end{array}$$

### Analysis results of sentiments in aspect-based feature extraction

Tables S15–S19 (http://doi.org/10.5281/zenodo.4444760) show the polarity scores of negative and positive sentiments including percentages of negative, positive, and neutral labels for STC, TAS, FGD, ATC, and STS datasets, respectively. Table 2 show the polarity scores for the STS dataset and it shows that all the categories were classified as negative, positive, and neutral tweets. Our proposed classification method gained a greater value of negative polarity score as 57.20% than positive polarity value as 23.60% in “Company” category. Furthermore, “Person”, “Movie” and “Product” categories have a high percentage in positive polarity labels as 64.80%, 78.90%, and 54%, whereas 19.60%, 5.30%, and 20.60% as negative polarity scores respectively. In “Location”, “Misc”, and “Event” categories, 49%, 42.60%, and 0% tweets were labeled as negative whereas positive polarity scores gained as 21.20%, 31.80%, and 82.85% respectively.

**Table 2:** Polarity scores with % of negative, positive and neutral labels in each category of STS dataset.

Sr No.	Category	Negative Scores (Polarity)	Positive Scores (Polarity)	Negative%	Positive%	Neutral%
1	Company	48.59	18.33	57.20	23.60	19.20
2	Person	9.6	26.67	19.60	64.80	15.60
3	Movie	1.69	8.89	5.30	78.90	15.80
4	Product	9.04	26.11	20.60	54	25.40
5	Location	7.91	1.67	49.00	21.20	29.80
6	Misc.	23.16	13.89	42.60	31.80	25.60
7	Event	0	4.4	0	82.85	17.15

### Analysis of hybrid aspect-based classification method

In this section, we present, analyze, and discuss the results of our proposed study. For each classifier, average results were considered of the proposed system. The performance of the different classifiers with feature selection techniques was compared. Different classification methods were used for the analysis and evaluation of the results obtained from the proposed approach. For this purpose, four different measures were used to evaluate the performance namely, Precision Eq. (5), Recall Eq. (6), Accuracy Eq. (7) and *F*-measure Eq. (8). In this proposed work, we ranked the features using IG to maximize the possibility of selecting meaningful features in the feature selection process as all the top-ranked features will get selected and PCA was used to select ranked features, and these features were used as input for feature classification method and MLP was used for classification purposes. Additionally, different classification methods were used to compare the results with the proposed technique. Four activation functions were implemented on all five datasets to evaluate the performance of MLP.

#### Discussion I

Table 3 shows the average results of the classification for STC dataset, Tables 4, 5, 6 and 7 show the average results for TAS, FGD, ATC, and STS datasets respectively, using 10 different approaches including K-Neighbors classifier, Decision Tree classifier, Logistic Regression, Support Vector classifier, Gaussian NB, Ada-Boost classifier, Gradient Boosting classifier, Random Forest classifier, Extra-Tree classifier and the classifier based on our proposed model i.e., the Multi-Layer Perceptron. Data in all these tables clearly show that a combination of MuLeHyABSC+MLP with POS tags achieved the highest accuracy. In contrast, Figures S1–S5 (http://doi.org/10.5281/zenodo.4444760), for all the five datasets respectively, demonstrates the average results of classification using the methods with which we compared our results and blend of MuLeHyABSC comprising of the Aspect-Based Sentiment Analysis(ABSA) plus using Sentiwordnet, PCA with the combination of MLP with POS tags.

**Table 3:** Analysis of proposed approach (MuLeHyABSC) for different approaches on testing model STC. The bold emphasis shows the highest results achieved by the proposed approach.

Sr. No.	Approach	Features	Accuracy (%)	Precision	Recall	*F*-score
1	MuLeHyABSC+MLP	POS tags + unigram	**78.99**	**0.79**	**0.789**	**0.789**
2	MuLeHyABSC+SVC	POS tags + unigram	75.79	0.758	0.757	0.757
3	MuLeHyABSC+LR	POS tags + unigram	72.08	0.712	0.722	0.716
4	MuLeHyABSC+DT	POS tags + unigram	73.02	0.725	0.732	0.728
5	MuLeHyABSC+KN	POS tags + unigram	70.16	0.714	0.721	0.717
6	MuLeHyABSC+RF	POS tags + unigram	72.08	0.712	0.722	0.716
7	MuLeHyABSC+AB	POS tags + unigram	75.34	0.755	0.753	0.752
8	MuLeHyABSC+ETC	POS tags + unigram	76.02	0.765	0.762	0.76
9	MuLeHyABSC+GB	POS tags + unigram	74.71	0.738	0.727	0.732
10	MuLeHyABSC+NB	POS tags + unigram	67.57	0.702	0.675	0.659

**Table 4:** Analysis of proposed approach (MuLeHyABSC) for different approaches on testing model TAS. The bold emphasis shows the highest results achieved by the proposed approach.

Sr No.	Approach	Features	Accuracy (%)	Precision	Recall	*F*-score
1	MuLeHyABSC+MLP	POS tags + unigram	**84.09**	**0.833**	**0.84**	**0.836**
2	MuLeHyABSC+SVC	POS tags + unigram	78.43	0.773	0.764	0.768
3	MuLeHyABSC+LR	POS tags + unigram	80.44	0.811	0.824	0.805
4	MuLeHyABSC+DT	POS tags + unigram	78.43	0.773	0.764	0.768
5	MuLeHyABSC+KN	POS tags + unigram	82.12	0.815	0.827	0.816
6	MuLeHyABSC+RF	POS tags + unigram	80.33	0.792	0.803	0.795
7	MuLeHyABSC+AB	POS tags + unigram	80.16	0.815	0.818	0.816
8	MuLeHyABSC+ETC	POS tags + unigram	81.58	0.802	0.815	0.804
9	MuLeHyABSC+GB	POS tags + unigram	80.33	0.792	0.803	0.795
10	MuLeHyABSC+NB	POS tags + unigram	72.43	0.733	0.714	0.721

**Table 5:** Analysis of proposed approach (MuLeHyABSC) for different approaches on testing model FGD. The bold emphasis shows the highest results achieved by the proposed approach.

Sr No.	Approach	Features	Accuracy (%)	Precision	Recall	*F*-score
1	MuLeHyABSC+MLP	POS tags + unigram	**80.38**	**0.794**	**0.803**	**0.793**
2	MuLeHyABSC+SVC	POS tags + unigram	76.06	0.755	0.77	0.747
3	MuLeHyABSC+LR	POS tags + unigram	74.65	0.723	0.746	0.702
4	MuLeHyABSC+DT	POS tags + unigram	72.86	0.731	0.724	0.727
5	MuLeHyABSC+KN	POS tags + unigram	74.31	0.721	0.743	0.689
6	MuLeHyABSC+RF	POS tags + unigram	74.31	0.721	0.743	0.689
7	MuLeHyABSC+AB	POS tags + unigram	71.06	0.726	0.702	0.713
8	MuLeHyABSC+ETC	POS tags + unigram	73.02	0.725	0.738	0.731
9	MuLeHyABSC+GB	POS tags + unigram	70.31	0.711	0.723	0.716
10	MuLeHyABSC+NB	POS tags + unigram	74.31	0.721	0.743	0.689

**Table 6:** Analysis of proposed approach (MuLeHyABSC) for different approaches on testing model ATC. The bold emphasis shows the highest results achieved by the proposed approach.

Sr No.	Approach	Features	Accuracy (%)	Precision	Recall	*F*-score
1	MuLeHyABSC+MLP	POS tags + unigram	**82.37**	**0.813**	**0.823**	**0.817**
2	MuLeHyABSC+SVC	POS tags + unigram	76.22	0.751	0.769	0.759
3	MuLeHyABSC+LR	POS tags + unigram	80.15	0.793	0.808	0.795
4	MuLeHyABSC+DT	POS tags + unigram	79.93	0.781	0.799	0.785
5	MuLeHyABSC+KN	POS tags + unigram	79.63	0.779	0.796	0.784
6	MuLeHyABSC+RF	POS tags + unigram	76.22	0.751	0.769	0.759
7	MuLeHyABSC+AB	POS tags + unigram	71.72	0.731	0.729	0.729
8	MuLeHyABSC+ETC	POS tags + unigram	80.24	0.785	0.802	0.789
9	MuLeHyABSC+GB	POS tags + unigram	79.93	0.782	0.799	0.786
10	MuLeHyABSC+NB	POS tags + unigram	71.72	0.731	0.729	0.729

**Table 7:** Analysis of proposed approach (MuLeHyABSC) for different approaches on testing model STS. The bold emphasis shows the highest results achieved by the proposed approach.

Sr No.	Approach	Features	Accuracy (%)	Precision	Recall	*F*-score
1	MuLeHyABSC+MLP	POS tags + unigram	**84.72**	**0.851**	**0.847**	**0.848**
2	MuLeHyABSC+SVC	POS tags + unigram	80.55	0.812	0.805	0.808
3	MuLeHyABSC+LR	POS tags + unigram	79.16	0.802	0.791	0.796
4	MuLeHyABSC+DT	POS tags + unigram	80.55	0.812	0.805	0.808
5	MuLeHyABSC+KN	POS tags + unigram	76.16	0.772	0.761	0.766
6	MuLeHyABSC+RF	POS tags + unigram	79.16	0.802	0.791	0.796
7	MuLeHyABSC+AB	POS tags + unigram	80.55	0.812	0.805	0.808
8	MuLeHyABSC+ETC	POS tags + unigram	72.55	0.732	0.725	0.728
9	MuLeHyABSC+GB	POS tags + unigram	80.55	0.812	0.805	0.808
10	MuLeHyABSC+NB	POS tags + unigram	66.66	0.683	0.666	0.674

According to the results shown in Table 3 of the STC dataset, the average highest accuracy was 78.99% of the proposed model MuLeHyABSC+MLP with POS tags and unigram features. Also, the precision, recall, and *F*-score were 0.790, 0.789, and 0.789 respectively. In the TAS dataset (see Table 4), the average highest values achieved for accuracy, precision, recall and *F*-score were 84.09%, 0.833, 0.840 and 0.836 respectively. Using the FGD dataset, the highest accuracy was measured as 80.38%, precision was 0.794, recall was 0.803 and *F*-score was 0.793 (see Table 5). Besides this, MuLeHyABSC+GB with the same features obtained the lowest accuracy as 70.31%, precision as 0.711, recall as 0.723, and F-measure as 0.716. In the ATC dataset (see Table 6), the highest accuracy was gained in MuLeHyABSC with the combination of MLP using POS tags + unigram features as 82.37%, precision, recall, and F-score measures were 0.813, 0.823, and 0.817 respectively. In the STS dataset (see Table 7), the highest achieved accuracy was 84.72%, precision was 0.851, recall and *F*-score measures were 0.847 and 0.848 respectively. In summary, these obtained results show that MLP performed better with POS tags + unigram feature set and proved it as an improved approach to classifying the tweets in each category so, MuLeHyABSC with the fusion of MLP achieved the highest accuracy in all the datasets. Achieved results for all the five datasets respectively show the average results of classification using the methods with which we compared our results and blend of MuLeHyABSC comprising of the Aspect-Based Sentiment Analysis(ABSA) plus using Sentiwordnet, PCA with the combination of MLP with POS tags.

### Evaluation of different activation functions

All five datasets were used to evaluate 24 iterations of MLP with different activation functions and size of layers. Different functions were tested to compare the results to achieve the best one. There is no hard and fast rule for choosing the best activation function. It depends on the architecture and the requirement of a model that gives optimal results. The average results are considered in this proposed work for each activation function and layer size after 24 iterations. These activation functions were used to evaluate the proposed system to attain the maximum accuracy. The purpose of these activation layers was to express the impact of neurons and layer sizes on the accuracy of the proposed model.

#### Discussion II

Multiple activation functions used in different deep learning methods were combined in this proposed approach and used in a single deep learning method MLP. We used four activation functions named as: Identity Eq. (1), Logistic or Sigmoid Eq. (2), Hyperbolic Tangent function (Tanh) Eq. (3) and Rectified Linear Units (ReLU) Eq. (4) used in Akhtar et al. (2017). In this proposed work, MLP architecture was designed to fit all the sizes of datasets independent of domain. The main step involved in this process was to analyze the test data. All the activation functions used in this approach compared the train feature set to the test feature set and obtained results of the classification through the output layer. The attributes of the datasets are proportional to the total number of neurons used in the input layer and polarity classes of the datasets depend on the total number of neurons considered in the output layer such as positive and negative. In the hidden layer, to minimize the error rate concerning data size, back-propagation was used while training the features to back-propagate again and further apply activation functions.

In this research work, the number of hidden layers (1–3) and neurons (50–100), were assigned according to the number of features that become input for the MLP model. In iterations 1, 2, 3, and 4, a total of 50 neurons were taken into account for testing purposes using 1 hidden layer, and all the mentioned activation functions were gradually applied on this layer. This hidden layer was fully connected with one output layer to classify the sentiments as positive and negative labels. In contrast, from iterations 5, 6, 7, and 8, we have used two hidden layers on top of each other with 50,50 neurons as 50 neurons in each layer for testing purposes. Besides this, in iteration 9, 10, 11, and 12, only one hidden layer was used with 100 neurons for testing purpose, incorporating four activation functions one at a time. On the other hand, in iteration 13, 14, 15, and 16, we have used two hidden layers interconnected with each other, with 100,100 neurons as 100 neurons in each hidden layer for testing purpose. Added to this, in iteration 17, 18, 19, and 20, we have considered three hidden layers on top of each other with 50, 50, 50 neurons as 50 neurons in each hidden layer for testing purpose. In contrast, iteration 21, 22, 23, and 24 used three fully connected hidden layers, with 100,100,100 neurons as 100 neurons in each hidden layer, and four activation functions were applied for testing purpose to classify the sentiments that were taken as input from the feature selection method using input layer. Besides this, 4 activation functions were applied one at a time for each iteration and the number of neurons mentioned above. Depicting 4 activation functions in the same layer elucidates that all the activation functions were applied gradually.

Some experimental results of the STC dataset are shown in Table 8 and detailed results of all 5 datasets are depicted in Tables S20–S24 (http://doi.org/10.5281/zenodo.4444760). In Table S20, maximum accuracy was achieved in iteration 5 using the “Identity” activation function, with two hidden layers on the top of each other and 50 neurons in each layer. Accuracy gained as 79.85%, precision, recall, and *F*-score as 0.799, 0.798, and 0.798 respectively. Besides this, TAS dataset results are depicted in Table S21, comprising of 24 iterations using 4 activation functions and maximum accuracy was achieved as 84.29%, with precision 0.834, recall 0.842 and *F*-score 0.837 in iteration 7 using “Tanh” activation function with two hidden layers and 50 neurons in each layer and same results were obtained in iteration 19 using activation function “Tanh” with three hidden layers and 50 neurons in each layer. Results of the FGD dataset are detailed in Table S22, maximum accuracy was achieved as 81.54% with precision, recall, and *F*-score as 0.795, 0.811, and 0.802 respectively in the 9th iteration using “Identity” activation function with one hidden layer of 100 neurons. Table S23 shows the results of the ATC dataset, maximum accuracy was obtained in the 4th iteration using the “ReLU” activation function with one hidden layer incorporating 50 neurons in it. Accuracy was achieved as 83.23%, precision as 0.828, recall as 0.832, and *F*-score as 0.829. Results of the STS dataset are shown in Tables S24 and maximum accuracy was achieved in the 4th iteration incorporating the “ReLU” activation function with 50 neurons in one hidden layer. In this table, the highest accuracy was achieved as 86.19%, precision, recall, and *F*-score as 0.875, 0.861, and 0.867 respectively. The ReLU activation function performed better in two datasets and obtained results were in an improved state during multiple iterations. In summary, an obvious feature of the proposed system reveals that even the most minimum accuracy value achieved by the proposed system was better than the results obtained from machine learning classification methods. These results show that the proposed system performed well in sentiment classification during each iteration using the deep learning method as results of machine learning approaches were below average as compare to these obtained results.

**Table 8:** Some experimental results of multiple iterations of activation functions of proposed approach (MuLeHyABSC) for STC. The bold emphasis shows the highest results achieved by the proposed approach.

Iterations	Activation function	Neurons	Accuracy (%)	Precision	Recall	F-measure
1	Identity	50,	77.25	0.775	0.772	0.773
2	Sigmoid	50,	78.99	0.789	0.789	0.789
3	Tanh	50,	79.25	0.792	0.792	0.792
4	ReLu	50,	76.88	0.769	0.768	0.768
5	Identity	50,50	**79.85**	**0.799**	**0.798**	**0.798**

### Existing benchmarks

In the previous research, PCA was used for improving the existing research results which were compared against the benchmarks LSA and RP. They compared the results of PCA with LSA and RP and showed that the accuracy of PCA was better than others. They used PCA+SVM as a hybrid approach to achieve values for accuracy, precision, recall and *F*-score as 74.24%, 0.751, 0.742 and 0.738 respectively, for STC dataset using POS tags + unigram and 76.5517% accuracy, 0.779 precision, 0.766 recall and 0.76 *F*-score for STS dataset using POS tags Zainuddin, Selamat & Ibrahim (2018) shown in Table 9. In contrast, Table S25 and Fig. S6 (http://doi.org/10.5281/zenodo.4444760) demonstrates the comparison of existing benchmark 1 with the proposed system for STC and STS datasets and resulted in improved accuracy for the proposed approach.

**Table 9:** Results of existing benchmark 1 for STC & STS datasets Zainuddin, Selamat & Ibrahim (2018). The bold emphasis shows the highest results of existing benchmark 1 for STC & STS datasets.

Approach	Features	Accuracy	Precision	Recall	*F*-Measure
ABSA+Sentiwordnet+PCA+SVM (STC)	POS tags+unigram	**74.2438**	**0.751**	**0.742**	**0.738**
ABSA+Sentiwordnet+PCA+SVM (STS)	POS tags	**76.5517**	**0.779**	**0.766**	**0.76**

In another paper (Go, Bhayani & Huang, 2009), the authors used SVM, NB and ME for training the dataset of Table S5 (http://doi.org/10.5281/zenodo.4444760), using different features unigram, bigram, and POS patterns. It gained maximum accuracy as 83% using MaxEnt with the combination of unigram + bigram features, using Naive Bayes with same features, got accuracy as 82.7% and using SVM with unigram + POS tags, it gained accuracy of 81.9% and results of existing benchmark 2 are shown in Table 10. We compared the results of the proposed system with the outcomes of these existing benchmarks. Figure S7 (http://doi.org/10.5281/zenodo.4444760) demonstrates that the proposed system achieved better results as compared to the outcomes of existing benchmark 2 in the STS dataset.

**Table 10:** Results of existing benchmark 2 for STS dataset Go, Bhayani & Huang (2009). The bold emphasis shows the highest results of existing benchmark 2 for STS dataset.

Features	Keyword	Naive Bayes	MaxEnt	SVM
Unigram + Bigram	N/A	**82.7**	**83**	81.6
Unigram + POS	N/A	79.9	79.9	**81.9**

**Figure 3:**
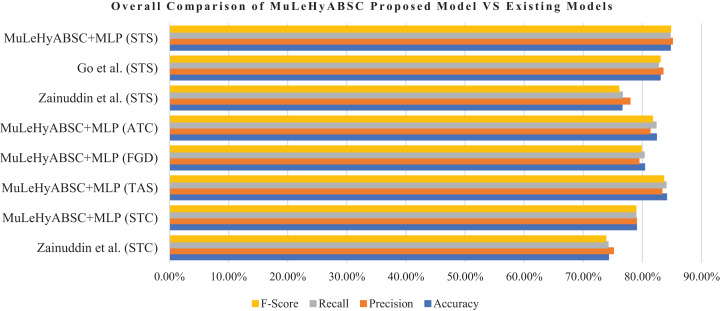
General evaluation of proposed model (MuLeHyABSC) with existing benchmark 1 Zainuddin, Selamat & Ibrahim (2018) & benchmark 2 Go, Bhayani & Huang (2009).

Table 11 shows the general comparison of proposed model (MuLeHyABSC+MLP) with the existing benchmark 1 Zainuddin, Selamat & Ibrahim (2018). The proposed system performed better for the STC dataset as compared to the existing benchmark 1 as they used PCA+SVM with POS tags + unigram and achieved an accuracy of 74.24% and the proposed system achieved an accuracy of 78.99% with precision, recall, and *F*-score as 0.79, 0.789 and 0.789 respectively. Furthermore, for the STS dataset, our proposed approach gained better results with an accuracy of 84.72%, with precision, recall, and *F*-measure as 0.851, 0.847, and 0.848 respectively. Whereas results of existing benchmark 1 for STS dataset, they achieved accuracy as 76.55%, precision as 0.779, recall as 0.766, and *F*-score as 0.76. Further, the detailed comparison of the proposed model with existing benchmark 2 (Go, Bhayani & Huang, 2009) is shown in Table S26 (http://doi.org/10.5281/zenodo.4444760). as they achieved the highest accuracy of 83% by using MaxEnt with unigram + bigram in the STS dataset and the proposed system achieved 84.72% accuracy by using MuLeHyABSC+MLP with POS tags + unigram and it showed improved results in an accuracy of predicted data. Tables S25 and S26 (http://doi.org/10.5281/zenodo.4444760) represents an individual comparison of the proposed system with existing benchmarks for STC and STS datasets. The results in Table 11 and Fig. 3 depicts that the proposed approach performed better in all types of datasets containing a different number of tweets.

**Table 11:** OVERALL comparison of MuLeHyABSC—proposed system’s achieved results with existing benchmarks. The bold emphasis shows the highest results achieved by the proposed approach.

Approach	Features	Accuracy (%)	Precision	Recall	F-measure
Existing Benchmark 1 (STC dataset)	POS tags + unigram	74.24	0.751	0.742	0.738
MuLeHyABSC+MLP (STC dataset)	POS tags + unigram	**78.99**	**0.79**	**0.789**	**0.789**
MuLeHyABSC+MLP (TAS dataset)	POS tags + unigram	**84.09**	**0.833**	**0.84**	**0.836**
MuLeHyABSC+MLP (FGD dataset)	POS tags + unigram	**80.38**	**0.794**	**0.803**	**0.798**
MuLeHyABSC+MLP (ATC dataset)	POS tags + unigram	**82.37**	**0.813**	**0.823**	**0.817**
Existing Benchmark 1 (STS dataset)	POS tags	76.55	0.779	0.766	0.76
Existing Benchmark 2 (STS dataset)	unigram + bigram	83.00	0.835	0.827	0.83
MuLeHyABSC+MLP (STS dataset)	POS tags + unigram	**84.72**	**0.851**	**0.847**	**0.848**

This research work can be useful for other languages as well, like in the English language tweet datasets, we used POS tags as adjectives, verbs, and adverbs to find more implicit aspects and determined sentiment words describing them as shown in the proposed system MuLeHyABSC model in Fig. 1. In this work, the proposed approach performed better with the fusion of deep learning method MLP in all the datasets (used in this work), whilst machine learning approaches used for classification purposes didn’t perform consistently, results varied in all the datasets. There are many possible reasons for inconsistency between results but one of the main reasons is variation in the sizes of datasets. In machine learning approaches, some classification methods performed better on small size datasets and some on large size datasets. In particular, POS tags + unigram features and the combination of IG with PCA in feature ranking and feature selection process resulted in performance gain in the deep learning method.

## Conclusions and future directions

The motivation of the proposed work was to perform finer-grained sentiment analysis to improve the functionality of aspect-based text classification using a hybrid approach. This study proposed an approach called: Multi-level Hybrid Aspect Based Sentiment Classification (MuLeHyABSC) comprising of multi-level (single and multi-word) aspect detection using ARM with the blend of heuristic POS patterns. The interconnection between noun phrases and heuristic mixture of POS patterns with verbs, adjectives, determiners, and adverbs was the main reason for valuable explicit aspects detection. Furthermore, the Stanford Dependency Parser (SDP) with the grammatical associations was used to find a relationship to extract implicit aspects including the determination of relations discovered by different types of dependencies. Our proposed approach also incorporates a feature ranking process by embedding a feature selection technique and further classification of sentiments using the deep learning method. This research aimed to use Twitter data to perform hybrid multi-level (single word and multi-word) aspect-based text classification. Different classification algorithms were implemented to compare the results with the proposed hybrid approach (MuLeHyABSC+MLP). The results showed that the proposed system for aspect-based text classification achieved significant improvement as compared to the existing baseline approaches proposed for sentiment classification by achieving accuracies of 78.99%, 84.09%, 80.38%, 82.37%, and 84.72% respectively. A neural network approach was used for large datasets which resulted in a performance gain. We plan to extend this research in the future by using some state-of-the-art approaches of ANN for aspect-based text classification. Latest feature extraction and feature selection techniques will be implemented with the combination of merged ANN methods including temporal aspects in the future for improving the existing system’s performance.

## Additional Information and Declarations

### Data Availability

The following information was supplied regarding data availability: Data is available at Zenodo: Sadaf Hussain Janjua, Ghazanfar Farooq Siddiqui, Muddassar Azam Sindhu, & Umer Rashid. (2021). Multi-level aspect based sentiment classification of twitter data: using hybrid approach in deep learning. DOI 10.5281/zenodo.4444760.
